# Can combined paravertebral and erector spinae block provide perioperative analgesia for mastectomy with LD flap reconstruction surgery? An observational study

**DOI:** 10.3332/ecancer.2024.1781

**Published:** 2024-09-27

**Authors:** Arunangshu Chakraborty, Sanjit Agrawal, Shiladitya Bose, Rosina Ahmed, Rakhi Khemka

**Affiliations:** 1 Department of Onco-Anaesthesia, Tata Medical Center, Kolkata 700160, India; 2 Department of Breast Onco-Surgery, Tata Medical Center, Kolkata, India; a https://orcid.org/0000-0002-0069-700X; b https://orcid.org/0000-0002-7631-655X

**Keywords:** ultrasound guided regional anaesthesia, breast cancer surgery, breast reconstruction, latissimus dorsi flap, perioperative analgesia

## Abstract

**Background:**

Mastectomy and breast reconstruction with latissimus dorsi myocutaneous flap (LDF) is a major surgery that covers eight or more dermatomes causing severe pain in the postoperative period.

**Objective(s):**

We evaluated the analgesic effect of a hybrid technique of ultrasound-guided combined thoracic paravertebral block (TPVB) and erector spinae plane block (ESPB) in a single needle pass in ten consecutive patients scheduled for mastectomy with LDF reconstruction as a part of a multimodal analgesia regimen.

**Design:**

Prospective observational study.

**Setting:**

A tertiary-level cancer hospital in Eastern India. The study was conducted between 01/09/2023 and 20/12/2023.

**Patients:**

10 consecutive consenting female patients of age between 18 and 75 years suffering from breast cancer, scheduled for a mastectomy with LDF reconstruction were recruited in this study, excluding patients with body mass index more than 40, coagulopathy or thrombocytopenia, skin conditions such as dermatitis, infection and so on, and known allergy to local anaesthetics (LAs).

**Intervention(s):**

The recruited patients received an ultrasound-guided combined thoracic paravertebral and erector spinae (COMPARES) block at the third thoracic (T3) level in a single needle pass, with 10 mL in the TPVB and 30 mL in the ESPB compartment, respectively, in a cephalad to caudad approach before induction of general anaesthesia.

**Main outcome measures:**

The primary endpoint was pain score at 9:00 am on postoperative day one. Other outcome measures were pain scores at postoperative hours 0 (immediately after awakening from general anaesthesia), 4, 8 and 12, postoperative nausea vomiting, requirement of rescue analgesics and pain score on shoulder movements on postoperative day one.

**Results:**

Median (range) resting pain scores at 0, 4, 8 and 24 hours were 1.5 (0–5), 2.5 (0–4), 2.5 (2–5) and 3 (2–4), and dynamic pain score on shoulder mobilization on postoperative day one morning was 3 (2–6). Only one patient required rescue analgesia.

**Conclusions:**

We found the technique inexpensive and potentially useful, but difficult in obese and short-statured patients due to increased depth and narrowing of the intertransverse space. This technique should be further evaluated in a randomised controlled trial.

**Trial registration:**

This trial was registered with the Clinical Trials Registry of India with the registration number CTRI/2023/08/057119.

## Key points

- Combined thoracic paravertebral and erector spinae block provides perioperative analgesia for mastectomy with latissimus dorsi myocutaneous flap (LDF) reconstruction
- While mastectomy involves anterior dermatomes of T2-4, the LDF involves dermatomes of T6–10, requiring additional analgesic management.
- The LDF area is innervated mostly by the dorsal rami of T6-10, rendering erector spinae plane block (ESPB) a suitable analgesic modality.
- 10 ml LA at the paravertebral (T3) and 30 ml LA at the ESPB in a single injection technique provides complete coverage of all the dermatomes.

## Introduction

**Figure 1.**
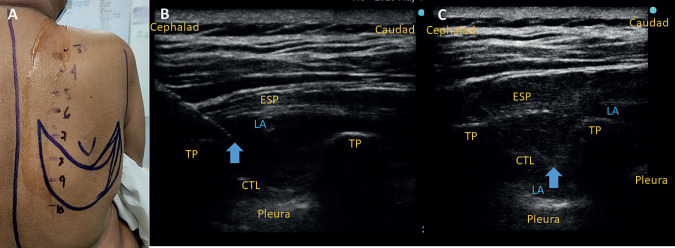
(A): Marking of the LD flap harvest site along with the thoracic dermatome levels. The flap harvest site corresponds to 6th to 10th thoracic (T6-T10) spine. (B): The ESPB at the 3rd thoracic level anterior and inferior to the 3rd thoracic TP. (C): The needle is further advanced to the paravertebral level beyond the superior costo-transverse ligament (CTL) and LA injected, which displaces pleura anteriorly as evidenced by the different levels of pleura in the adjacent spaces. ESP: Erector spinae muscle.

Breast cancer is the most common cancer in women and contributes to a large disease burden globally. Patients who require mastectomy for management of breast cancer are offered primary reconstruction, which may be autologous or implant based. Although free tissue transfer is the gold standard for autologous reconstruction, it is time consuming and expensive for patients. Whole breast reconstruction using a pedicled Latissimus Dorsi myocutaneous flap (LDF) was first described in the 1970s, and remains a reliable, versatile and widely used technique. LDF is a major surgery taking about four hours, involving 6th to 10th posterior thoracic dermatomes in the back for harvesting the graft (Figure 1) and second to fourth anterior thoracic dermatomes for the mastectomy and axillary clearance. It causes severe postoperative pain and the patients would naturally benefit from regional anaesthesia.

While the preferred mode of analgesia for mastectomy has been discussed in volume, there are no guidelines for analgesia management for LDF reconstruction surgery. Thoracic paravertebral block (TPVB) has been successfully used for perioperative analgesia for breast surgery [1]. Although TPVB provides analgesia for about 10 hours, it has limited dermatomal coverage as injection at each level blocks the ventral roots of the spinal nerve above and below the level of injection. Injection at two levels is needed for adequate analgesia for breast surgery which typically involves the T2-T5 dermatomes [2]. LDF reconstruction surgery involves harvesting of a part of LD muscle from the back, (Figure 1) innervated mostly by the dorsal root of the spinal nerves of T6-10 dermatomes. The erector spinae plane block (ESPB) is a technique of ultrasound-guided regional anaesthesia (USRA) that deposits LA in the fascial plane below the erector spinae muscle. This technique addresses the dorsal roots more efficiently and to some extent the ventral roots also through anterior translocation and diffusion of the local anesthetic (LA) solution. The ESPB being a fascial plane block can cover a significantly larger number of dermatomes, depending on the volume injected [3]. We postulated that by combining TPVB and ESPB, one can achieve a combination of dense intraoperative analgesia and prolonged postoperative analgesia, while covering the entire dermatomes for a mastectomy with LDF reconstruction surgery.

All the patients consented to the scientific publication of the findings of this study. This manuscript adheres to the applicable EQUATOR guidelines.

## Methods

We conducted a single-arm prospective observational study of a combined paravertebral and erector spinae block (COMPARES) technique in 10 consecutive consenting patients scheduled for mastectomy, axillary clearance and breast reconstruction with LDF to characterize the perioperative analgesic effect.

**Ethics:** Ethical approval for this study was provided by Tata Medical Center-Institutional Review Board (TMC-IRB), India, (Chairperson Prof Siddhartha Roy) on 20.06.2023 (IRB approval # 2023/TMC/273/IRB47). This study was registered prospectively with the Clinical Trials Registry of India (CTRI/2023/08/057119).

Patients scheduled for mastectomy with LDF reconstruction surgery for breast cancer of age between 18 to 75 years were included, while patients with body mass index (BMI)>40, Coagulopathy or thrombocytopenia, Skin conditions such as dermatitis, infection and so on, and known allergy to LA were excluded. Ten patients were recruited between 01/09/2023 and 20/12/2023.

After taking to the operation theatre, establishing intravenous access and connecting standard monitors, the patients were asked to sit. With applicable aseptic precautions, an ultrasound scan of the back was performed in a sitting position, 3rd to 8th ribs and costotransverse junctions was identified and marked with a sterilised skin marker pen close to the costotransverse junction. A curvelinear or linear array ultrasound transducer (UST) was selected depending on the patients BMI. 2% lignocaine with epinephrine was infiltrated at the point of entry and an ESPB was performed under ultrasound guidance in-plane, with the needle-directed caudad at the level of the 3rd thoracic transverse process (TP) with 20 ml 0.2% ropivacaine. The block needle was redirected as necessary and further advanced in-plane to perform a TPVB at the 3rd TPV space with 10 ml of 0.2% ropivacaine. TPVB was confirmed with sonographic evidence of anterior displacement of the pleura and the parasagittal extent of the spread of LA in the ESPB was noted with ultrasound (Figure 1).

In obese patients, particularly with short stature, where the intertransverse gap is lesser than usual, we found it prudent to put the TPVB first, with 10 ml 0.2% ropivacaine and withdraw the needle consequently to the ES plane to inject another 30 ml. This approach required fewer redirections and took less time. The same anaesthesiologist (first author) administered all the blocks.

After administration of the block, the patients were preoxygenated and observed for 10 minutes for any haemodynamic change. General anaesthesia was administered with intravenous propofol 2 mg·Kg^_1^, fentanyl 2 mcg·Kg^-1^, cisatracurium 0.1 mg·Kg^-1^ and an armored tube of appropriate size was inserted. Anaesthesia was maintained with inhaled sevoflurane in an oxygen and air mixture with a bispectral index (BIS) of less than 50. Patients were ventilated to normocapnea. Intraoperative hemodynamic parameters were noted. All patients received ondansetron and dexamethasone near the end of surgery. Pain scores (numerical rating scale, NRS) in the postoperative period were noted at 0, 4, 8, 12 and 24 hours from the end of the surgery. As part of multimodal analgesia, all patients received intravenous paracetamol 1 gm 8 hourly and diclofenac sodium 75 mg 12 hourly. Whenever the pain score exceeded 4, the patients were administered a bolus of intravenous fentanyl 0.5 mcg·Kg^-1^ and an intravenous patient-controlled analgesia (PCA) pump with fentanyl was provided.

The primary outcome was pain score (NRS 0–10) at 9:00 AM on POD 1 and the secondary outcomes were Perioperative requirement of rescue analgesics, pain scores (NRS 0–10) at-immediately after (0), at 4 and 8 hours after emergence from anaesthesia and incidence of postoperative nausea and vomiting (PONV).

To reduce reporting bias, the pain scores were taken and entered in the case report form by post anaesthesia care unit (PACU) nurses in the PACU and acute pain unit nurses in the ward.

### Results

Intraoperatively, none of the patients needed additional opioids and all of them exhibited a stable haemodynamics and electroencephalogram (EEG) pattern with BIS consistently below 50. Median (range) pain scores (NRS) at 9 AM, POD 1 and 0, 4, 8 and 24 hours were 3 (2–4), 1.5 (0–5), 2.5 (0–4), 2.5 (2–5) and 3 (2–4) (Table 1).

Only one patient required rescue analgesic and PCA. The same patient complained of PONV. The rest of the nine patients reported excellent analgesia in the postoperative period. All of them had a complete range of shoulder mobility with dynamic pain scores <4 (NRS) observed by the surgical team in the morning rounds on postoperative day one (POD1). None of the patients had any block-related complications. The cost of the single shot ultrasound-guided block including the ultrasound reflecting blunt-tipped needle, LAs and sterile equipment such as probe cover, gloves and so on (Ultrasound usage is not charged) amounted to INR 2000/-(USD 24.11), which was 255% lesser than a continuous TPVB/ESPB infusion and 10% lesser than a fentanyl PCA for 24 hours.

## Discussion

LDF reconstruction surgery along with mastectomy is a major surgery involving 7–8 dermatomes, (Figure 1) yet, neither a specific guideline, nor much clinical evidence exists regarding perioperative analgesia for this surgery. The PROSPECT group recommends TPVB for breast oncological surgery but does not specify anything for LD reconstruction [4].

Unkart *et al* [5] in a retrospective review, compared single shot versus continuous TPVB for LDF reconstruction surgery, and the average pain score in their limited series was 4.4 and 3.5 in the single shot and the continuous TPVB groups, respectively. In our series, the median (range) pain score at 24 hours was 3 (2–4), that too with a single shot technique.

The prevalent description of ultrasound-guided TPVB in the parasagittal approach entails the entry of the block needle in a caudal to cephalad direction, while the technique of ESPB, on the other hand, requires the needle direction from cephalad to caudad. We tried combining these two techniques in a single needle pass in a cephalad to caudad direction. Our aim was to devise a method by which we could maximise analgesic effect with only one needle pass, which is why we chose the 3rd intertransverse space for the TPVB as it could cover the principal two dermatomes, i.e., T3 and T4 innervating the breast area. We chose the cephalad to caudad direction as we put more emphasis on the ESPB and caudad spread of the injectate from the site of injection to reach the lower dermatomes and thus cover the area of the LDF graft which contributes more to the overall postoperative pain. We chose the volume of ESPB as 30 ml so that the LA could reach the T10 level, the distal-most dermatome involved in the LDF reconstruction surgery.

We observed the patients for up to 24 hours as most of our LDF graft patients, unless complicated, get discharged on POD1. The analgesia and ease of shoulder mobilisation helped the surgical team to discharge these patients early.

While the ESPB is the easier block, in our experience, a considerable degree of redirection and often fresh needle pass is required to reach to paravertebral space unless the original angle of entry is close to a perpendicular. It was more difficult in patients with obesity and short stature. An initial estimate of the depth of the paravertebral space helps in selecting the UST, point and angle of entry. ‘More the depth, more the angle of entry’ can be a useful dictum. The initial target should be the lower edge of the upper TP, grazing which, the needle can then advance to the paravertebral space. A point of entry about 1.5 cm away from the footprint of the UST is beneficial as it can improve needle visualisation. Incremental hydrodissection can be useful, particularly in obese patients. Another plausible advantage of this technique can be the creation of a potential channel of LA translocation from the erector spinae plane to the paravertebral space. Although it is purely a hypothesis now, it should be investigated via imaging and cadaveric studies.

While the effects of opioids on cancer recurrence [6] is being studied and reports indicate the protective effect of LA against cancer recurrence [7], possibly the paucity of the number of cases precludes clinical research in the domain of perioperative analgesia for major breast reconstruction surgeries. Including a regional anaesthesia component in the bouquet of multimodal analgesia not only reduces opioid consumption and opioid-related side effects, but also reduces chronification of acute pain [8] and improves shoulder mobility [9] after breast surgery.

Zengin *et al* [10] used combined paravertebral and erector spinae (COMPARES) plane block for video-assisted thoracoscopic surgery successfully and reported effective postoperative analgesia. To the best of our knowledge, this is the first report of a COMPARES technique used for mastectomy and axillary clearance with LDF reconstruction. COMPARES can provide postoperative analgesia for up to 24 hours at a low cost.

One major limitation of our study was that we could not perform dermatomal mapping due to time constraints before surgery and the presence of the surgical dressing afterward. We understand that it is only an initial report and it needs to be further evaluated in a randomised controlled setting against no block, LA infiltration, only TPVB and only ESPB.

## Experience from India

The availability of opioids in India is abysmally low, with less than 1% of the people in need having access to opioids. Clinicians in most of hospitals have to rely on weaker analgesics such as non-steroidal anti-inflammatory drugs, paracetamol and weaker opioids such as tramadol and pentazocine. As a result, the patients’ pain remains poorly controlled. Postoperative pain is generally ignored by the medical fraternity and patients are encouraged to tolerate pain with advises such as ‘pain after surgery is normal.’ With a severe shortage of manpower, only a few hospitals have round-the-clock acute pain service and the luxury of having a pain nurse.

Intravenous PCA with morphine or fentanyl, which is the standard of care for postoperative analgesia for breast reconstruction surgeries in many high-income countries, is available only in a handful hospitals in India, most of which are private ones, where the cost of treatment is high and often out of the reach of the common men. Over the last decade, the availability of Ultrasound has improved in India, bringing in an era of USRA albeit later than the developed world. Once the machine is available, USRA provides a safe and inexpensive modality of perioperative pain management through single shots and continuous nerve blocks. USRA is not a common practice in breast surgery in India. We had to discuss with the surgical team, conduct a few blocks on a pilot basis and present the merits of the modality before we could agree on a prospective trial. The anaesthesia department having clinical fellows and a pain nurse helped us conduct the study.

COMPARES holds a special promise in India and other developing economies where ultrasound is available in the operation theatre. Apart from being an excellent analgesic modality, it provides advantages over PCA in terms of cost, opioid sparing and compliance with enhanced recovery after surgery protocols. The encouraging reports of this prospective observational trial should inspire randomised controlled trials to further evaluate the analgesic efficacy of COMPARES for LDF and other breast reconstruction surgeries using myocutaneous or fasciocutaneous flaps, in comparison with no block as well as other regional anaesthesia modalities such as segmental spinal analgesia, TPVB and ESPB.

## Conclusion

We found the COMPARES technique inexpensive and potentially useful for perioperative analgesia in LDF surgery, but difficult in obese and short-statured patients due to increased depth and narrowing of the intertransverse space. This technique should be further evaluated in a randomised controlled trial.

## List of abbreviations

COMPARES, combined paravertebral and erector spinae plane block; ESPB, erector spinae plane block; TPVB, thoracic paravertebral block; LA, local anesthetic; LD, latissimus dorsi muscle; LDF, latissimus dorsi myocutaneus flap; UST, ultrasound transducer.

## Author contributions

Dr. Arunangshu Chakraborty: This author conceived and conducted the study and wrote the manuscript. Dr. Sanjit Agrawal: This author helped in designing the study and patient recruitment and reviewed the manuscript. Dr. Shiladitya Bose: This author helped with patient recruitment and data collection. Dr. Rosina Ahmed: This author helped with patient recruitment and reviewed the manuscript. Dr. Rakhi Khemka: This author helped with the study design, conduct and reviewed the manuscript.

## Conflicts of interest

The authors declare no conflicts of interest.

## Funding

None declared.

## Presentation

None declared.

## Figures and Tables

**Table 1.** Demographic and descriptive characteristics.

Parameter (units)	Indicator	Values
Age (years)	Median (range)	42 (19–62)
BMI	Median (range)	25.6 (21.34–33.9)
ASA PS	Median (range)	1 (1–2)
Duration of surgery (minutes)	Median (range)	215 (180–330)
Post-operative pain scores (NRS) at 9 AM, POD 1	Median (range)	3 (2–4)
Post-operative pain scores (NRS) at 0 hour	Median (range)	1.5 (0–5)
Post-operative pain scores (NRS) at 4 hours	Median (range)	2.5 (0–4)
Post-operative pain scores (NRS) at 8 hours	Median (range)	2.5 (2–5)
Post-operative pain scores (NRS) at 24 hours	Median (range)	3 (2–4)
Rescue analgesia	n (%)	1 (10%)
PONV	n (%)	1 (10%)
Pain score on shoulder movement on POD1	Median (range)	3 (2–6)

*ASA PS: American Society of Anesthesiologists Physical Status
